# Recent updates in the treatment of diabetic polyneuropathy

**DOI:** 10.12703/r/11-30

**Published:** 2022-10-18

**Authors:** Qihua Fan, A Gordon Smith

**Affiliations:** 1 Department of Neurology, Division of Neuromuscular Medicine, Virginia Commonwealth University, Richmond, VA, USA

**Keywords:** diabetic polyneuropathy, biomarkers, obesity, metabolic syndrome, neuropathic pain, genetic modifiers, therapeutics

## Abstract

Distal symmetric diabetic peripheral polyneuropathy (DPN) is the most common form of neuropathy in the world, affecting 30 to 50% of diabetic individuals and resulting in significant morbidity and socioeconomic costs. This review summarizes updates in the diagnosis and management of DPN. Recently updated clinical criteria facilitate bedside diagnosis, and a number of new technologies are being explored for diagnostic confirmation in specific settings and for use as surrogate measures in clinical trials. Evolving literature indicates that distinct but overlapping mechanisms underlie neuropathy in type 1 versus type 2 diabetes, and there is a growing focus on the role of metabolic factors in the development and progression of DPN. Exercise-based lifestyle interventions have shown therapeutic promise. A variety of potential disease-modifying and symptomatic therapies are in development. Innovations in clinical trial design include the incorporation of detailed pain phenotyping and biomarkers for central sensitization.

## Introduction

Diabetes mellitus is a worldwide pandemic, affecting 537 million adults^1^ (37.3 million in the US as of 2021^2^), a number that is projected to increase to 783 million people by 2045^3^. Diabetic peripheral polyneuropathy (DPN) affects about 50% of patients with diabetes mellitus and is the most common cause of neuropathy worldwide^4,5^. DPN is also the precipitating risk factor for diabetic foot complications, including diabetic ulcers, Charcot arthropathy, and lower limb amputations. These sequelae are independently associated with increased mortality risk^6^. Associated healthcare costs related to diabetes mellitus and its complications increased from $232 billion in 2007 to $760 billion in 2019 worldwide^7^, and up to 27% of these costs are attributed to DPN^8^.

Diabetic neuropathies may be classified into generalized and focal/multifocal forms. The most common diabetic neuropathy is a length-dependent, symmetrical sensory-motor peripheral polyneuropathy^9^. DPN develops in the context of a system of metabolic derangements, including hyperglycemia, increased polyol flux, oxidative stress, and lipid alterations, in addition to other cardiovascular risk factors^9–12^. The Toronto consensus criteria provide a framework for DPN diagnosis, which is based on the combination of neuropathy symptoms and signs and can be confirmed using nerve conduction studies (NCSs)^13^. NCSs are normal in small fiber neuropathy, which is usually associated with significant neuropathic pain. In this setting, a validated measure of unmyelinated small-diameter axonal injury may be used to confirm the diagnosis^14^. The most used diagnostic tool for small fiber neuropathy is a skin biopsy with quantification of intraepidermal nerve fiber density (IENFD)^14^.

In this article, we highlight five areas of recent updates in DPN: evolving biomarkers for early and accessible diagnosis, metabolic risk factors, innovations in clinical trials for painful diabetic neuropathy, genetic modifiers of disease risk, and recent therapeutic developments.

## Novel diagnostic tests and biomarkers

A major criticism in the screening process for DPN is that by the time neuropathy becomes detectable by current assessments; nerve injury is well established and difficult to reverse^15,16^. Thus, there is a need for more increasingly sensitive and responsive biomarkers as screening and diagnostic tools and surrogate end-point measures^17^. Accurate diagnosis of different DPN phenotypes, including small fiber neuropathy, is necessary for clinical trial design and to facilitate targeted therapeutic intervention. The 2020 Analgesic, Anesthetic, and Addiction Clinical Trial Translations, Innovations, Opportunities, and Networks (ACTTION) criteria are standardized diagnostic criteria for idiopathic large, small, and mixed fiber neuropathies for research use: at least one small or large fiber symptom and sign and abnormal IENFD or sensory NCS (or both) for small fiber, large fiber, and mixed polyneuropathies, respectively^18^. Publication of specific aligned criteria for DPN is expected in 2022. IENFD remains the gold standard pathological confirmation of small fiber neuropathy, but the test is minimally invasive, and the biopsy specimen needs to be processed and evaluated at highly experienced laboratories to avoid false-positive results that can occur from suboptimal handling^18^. The development of less invasive and more easily performed biomarkers is necessary to facilitate diagnosis and design of clinical trials for disease prevention or early intervention. A summary of the biomarkers discussed below is shown in Table 1.

**Table 1.** Summary of novel biomarkers in diagnosis and evaluation of diabetic polyneuropathy.

Biomarker	Advantages	Obstacles
Corneal confocal microscopy	Noninvasive	Requires specialist training and equipment Lack of normative data
Nerve excitability testing	Reversible changes Noninvasive	Requires specialist training and equipment More reliable in motor than sensory nerves No information about small fibers Lack of normative data
Microneurography	Minimally invasive May be helpful in identifying treatment-responsive pain phenotypes	Requires specialist training and equipment Not specific to neuropathic pain Lack of normative data
Neurofilament light chain	Blood biomarker myelin protein zero (MPZ) mRNA levels predict future axon loss 24 months in advance and predict hypo- vs. hyperalgesic phenotype	Nonspecific
Inflammatory markers	Blood biomarker Associated with risk of developing diabetic peripheral polyneuropathy (DPN); may be more helpful in risk stratification than drug target	Nonspecific
Functional magnetic resonance imaging (fMRI) as a biomarker for pain	Demonstrates central nervous system (CNS) role in sensitization and deafferentation May be helpful in identifying treatment-responsive pain phenotypes	Requires specialist training and equipment Lack of normative data

Corneal confocal microscopy is a noninvasive technique that can detect and quantify small nerve fiber loss in DPN and other forms of neuropathy^19^. A confocal laser scanning microscope noninvasively visualizes small-diameter unmyelinated axons in the cornea. Patients with DPN have reduced corneal nerve fiber density and length compared with normal controls^19^. In a cohort of 143 patients with diabetes^20^, corneal fiber density correlated with neuropathy signs on examination. Comparison of corneal nerve fiber length and density against neuropathy exam findings (vibration, cold, and warmth sensation thresholds) in controls (n = 30), painful diabetic neuropathy (n = 78), and painless diabetic neuropathy (n = 62)^21^ suggested that inferior whorl fiber changes preceded changes at the central whorl in a length-dependent fashion and correlated with decreased cold and warmth perception thresholds in those with painful diabetic neuropathy^21^. More investigation and validation studies are needed before corneal confocal microscopy can be considered as an alternative measure of small-caliber nerve fiber loss.

Nerve excitability testing (NET) may show promise as an emerging experimental neurophysiological biomarker of early axonal dysfunction. NET measures axonal firing thresholds in response to submaximal and supramaximal current delivered via noninvasive electrodes^22^, acting as a surrogate of axonal membrane dysfunction before axonal damage occurs and NCS findings are evident^23,24^. The hope is that NET may detect early changes in axonal function in DPN before axonal degeneration becomes irreversible, as demonstrated in oxaliplatin-induced neurotoxicity^25^. Patients with mild DPN (absent H-reflexes or distal sensory nerve conduction slowing) had abnormal axonal excitability profiles compared with healthy controls: longer duration of the relative refractory period, lesser prominent change of superexcitability, and smaller threshold changes to 50% depolarizing current^26^. Limitations of this technology include (1) this technique requires specialist training and equipment and is not widely available, (2) NET is more reliable and reproducible in motor nerves than sensory nerves^27^, (3) it does not provide information about the status of small fiber nerves, (4) no clinically relevant normative ranges are defined, and (5) NET still needs to be validated as an alternative biomarker for diabetic peripheral neuropathy^22^.

Neurofilament light chain (NfL) protein, a marker of axonal degeneration, and circulating myelin protein zero (MPZ) mRNA transcripts show promise as blood biomarkers for diabetic neuropathy^28^. NfL is increased in DPN patients compared with controls, and MPZ mRNA transcript levels are reduced^28^. The latter may predict future axonal loss 24 months in advance^28^. Reduced MPZ mRNA levels predicted a hypoalgesic phenotype as opposed to increased NfL levels, which predicted a hyperalgesic phenotype^28^. NfL has been proposed as a biomarker of many neurodegenerative conditions, including motor neuron disease, degenerative movement disorders, dementia, and hereditary amyloidosis^29^. Serum NfL levels have also been shown to correlate with treatment response in patients with hereditary transthyretin amyloid (variant transthyretin amyloidosis, or ATTRv) polyneuropathy^30^. Patients with ATTRv polyneuropathy had higher levels of serum NfL than healthy controls (16 vs. 69.4 pg/mL, respectively), and after 18 months, serum NfL levels increased with placebo (36.3 mg/mL increase) and decreased with transthyretin (TTR) silencer patisiran treatment (-23.3 pg/mL), correlating with clinical progression in the placebo group versus the treatment group^30^. While nonspecific, this correlation with treatment response suggests that NfL holds promise as a biomarker for disease progression and treatment response in neurodegenerative diseases, including DPN.

In contrast with diabetic patients without neuropathy, patients with DPN in type 2 diabetes showed elevated inflammatory markers C-reactive protein (CRP), tumor necrosis factor-alpha (TNF-α), intercellular adhesion molecule 1 (ICAM-1), and interleukin 6 (IL-6)^17,31^. High levels of TNF-α and IL-6 were associated with the development of DPN over time^32^. An increase in systemic inflammatory markers TNF-α and IL-6 was associated with DPN in type 2 diabetes, but these markers were also increased in painful neuropathies of various etiologies over nonpainful neuropathies^31^. Cytokine activation is associated with the generation of neuropathic pain^33^, but cytokine-specific antagonists (such as TNF-α inhibitors) failed to treat neuropathic pain^34^, suggesting that cytokines and chemokines function more as a network than single proteins in mediating painful polyneuropathies^31^.

There is increasing evidence that changes in the brain and spinal cord reflect and modulate neuropathic pain in DPN^35–38^. In animal models, interventions that enhance or reduce spinal inhibition resulted in respectively decreased^35,39^ or increased^40^ behavioral indices of pain^41^. Additionally, advanced imaging shows cortical changes that may serve as promising biomarkers in painful DPN, which we will discuss below.

## The role of obesity and the metabolic syndrome in DPN pathogenesis

While both type 1 and type 2 diabetes are characterized by hyperglycemia, type 1 diabetes is caused by autoimmune injury to pancreatic beta cells resulting in reduced insulin and C-peptide levels^42^, whereas type 2 diabetes is due to insulin resistance in association with metabolic risk factors, including obesity and dyslipidemia^43^. Multiple pathophysiologic pathways contribute to the development of diabetic peripheral neuropathy, including microvascular injury and ischemic stress to the peripheral nerve, inflammation, oxidative stress, and mitochondrial injury^41^. Multiple animal studies and cross-sectional, observational, and case-control studies across multiple continents spanning 40 years strongly suggest that metabolic syndrome and its component features are associated with an increased risk of developing polyneuropathy in type 2 diabetes and idiopathic neuropathy as well as in long-standing type 1 diabetes^44^. Metabolic syndrome is defined by the presence of at least three out of five criteria: elevated serum triglycerides, reduced high-density lipoprotein cholesterol, central obesity, hypertension, and diabetes or prediabetes^45^.

Both idiopathic peripheral polyneuropathy and diabetic polyneuropathy manifest with length-dependent sensory loss, with preferential injury to small nerve fibers. In addition, the presence of metabolic syndrome is a significant risk factor for developing polyneuropathy in both type 1 and type 2 diabetes^44^. The association between metabolic syndrome and sensory polyneuropathy is present independent of glycemic status in multiple studies^46–49^, suggesting that the other components of the metabolic syndrome may play a greater role in modulating the development of neuropathy in type 2 diabetes. In type 1 diabetes, aggressive glucose control significantly reduces the risk of neuropathy, but this effect is much more attenuated in type 2 diabetes (the relative risk reduction in type 1 is over 75% but is less than 10% in type 2 diabetes)^50,51^. Both dyslipidemia and prediabetes are also independently associated with the development of idiopathic peripheral polyneuropathy^44^. A meta-analysis investigating the association of dyslipidemia and diabetic neuropathy in 2021 examined 39 clinical trials containing 32,668 patients with either type 1 or type 2 diabetes and found that higher triglyceride and low-density lipoprotein levels were associated with an increased risk of diabetic neuropathy^52^. Tissue-specific dyslipidemia profiles distinguished diabetic nephropathy, retinopathy, and neuropathy, each with a distinct set of lipid species affected^53^. In a diabetic mouse model, overall lipid species were increased in kidneys and nerves, while lipid content was decreased in retinas. In human sural nerve biopsies, progressive and stable diabetic neuropathies had distinct transcriptomic profiles^53,54^. Of the three diabetic complications, diabetic neuropathy is most associated with dyslipidemia, which in turn induces mitochondrial deficits and accumulation of lipotoxic species to axons leading to axonal degeneration^55^.

Growing evidence suggests that obesity alone without hyperglycemia is a significant risk factor for neuropathy^56^. More individuals with obesity had neuropathy compared with lean individuals, and the presence of neuropathy has been associated with abdominal obesity, hypertension, and elevated triglycerides^56^. Additionally, obese patients without neuropathy had reduced intraepidermal nerve fiber densities and worse pain, quality of life, and depression scores in contrast with lean controls^56,57^.

The impact of aggressive management of metabolic syndrome features has yet to be determined; however, clinical and preclinical data suggest that this approach may have promise. Lifestyle interventions over 12 weeks, including strategies for glucose control, physical activity, weight loss, and diet modifications, reduced the severity of DPN symptoms^58^. Similarly, the Look AHEAD study demonstrated that intensive lifestyle modifications significantly decreased DPN symptoms, which were associated with a degree of weight loss 1 to 2.3 years after termination of active intervention^59^. Multiple clinical trials are under way to examine the effect of lifestyle interventions on diabetic neuropathy (NCT04813146 and NCT01565317). The Topiramate as a Disease Modifying Treatment for Cryptogenic Sensory Neuropathy (TopCSPN) trial (NCT02878798), funded by the National Institutes of Health, is examining whether 100 mg of topiramate daily slows the progression of idiopathic neuropathy based on weight loss and its effects on metabolism. Results are expected later this year.

## Innovations in painful DPN clinical trial design

About 30% of patients with DPN have neuropathic pain, which is a significant cause of patient morbidity^60–62^. There is increasing interest in whether the pain phenotype may help characterize the underlying pathophysiology and thereby suggest a more tailored treatment approach. Characterization by quantitative sensory testing (QST) of over seven types of neuropathic pain syndromes (including polyneuropathy, postherpetic neuralgia, peripheral nerve injury, trigeminal neuralgia, and central pain) showed that the pain phenotypes were incredibly heterogeneous and that different patients with the same disease can have different phenotype profiles (such as pinprick hyperalgesia vs. hypoalgesia)^63^. In studies characterizing subjective pain descriptors, two types of neuropathic pain have been described: ongoing burning pain and electric shock-like sensations^64^. While there was no clear relationship between burning pain and specific patterns of abnormal sensory modalities on QST, the level of burning pain was inversely related to laser-evoked potentials, which primarily measure Aδ fibers^64^. In contrast, electric shock-like sensations were associated with abnormal non-nociceptive Aβ-fibers based on somatosensory-evoked potentials or NCS abnormalities^64^. Four theoretical mechanisms^61^ underlie ongoing burning neuropathic pain: (1) sensitization of “irritable” nociceptors where distal nerve terminals are spared and IENFD may be normal, (2) hyperexcitable “regenerating sprouts” in ongoing regeneration with reduced IENFD, (3) functional deafferentation due to distal axonal degeneration manifesting as distal numbness and proximal hypersensitivity, and (4) anatomical denervation in processes such as ganglionopathy or root lesions where the pain is felt in the same region as hypoesthesia^64^.

Sodium channel blockers have been explored as a potentially efficacious therapy for the irritable nociceptor phenotype^65^. Demant *et al.* (2014) categorized 97 patients with neuropathic pain^64^ as having an irritable nociceptor versus nonirritable phenotype^66^. These patients were randomly assigned to treatment with oxcarbazepine or placebo. The numbers needed to treat for a more than 50% reduction in total pain score were 3.9 in the irritable nociceptor group and 13 for the nonirritable nociceptor group^66,67^, suggesting that sodium channel antagonists may be effective in the irritable nociceptor phenotype.

Microneurography may serve as a biomarker for the irritable nociceptor phenotype and as an objective, quantifiable measure of subjective pain^68^. In this technique, a microelectrode is inserted into the nerve fascicle and records action potentials from a single axon, thus measuring the degree of spontaneous activity from the peripheral nerve. Patients with painful polyneuropathy demonstrate a higher proportion of spontaneously active or mechanically sensitized C-nociceptors in contrast to patients with painless polyneuropathy and also showed less activity-dependent slowing suggestive of a peripheral sensitization^69^. However, microneurography is a time-consuming process performed at a few institutions and requires special expertise by the investigator and collaboration from the awake patient. Furthermore, there are no normative data in healthy subjects, and C-type nociceptor hyperactivity and sensitization may not be specific to patients with peripheral neuropathy, as this has also been demonstrated in patients with other etiologies of pain, including fibromyalgia^70^, erythromelalgia^71^, or complex regional pain syndrome^72^. While these limitations prohibit its use in bedside clinical practice, it may be a promising option in identifying the subset of patients with irritable nociceptor phenotype that might be more responsive to treatment, demonstrated in the randomized control trial of ABT-639 (a T-type calcium channel blocker) on spontaneous C-type nociceptor activity in patients with painful diabetic neuropathy^73^. Although the trial showed no differences in C-nociceptor activity or pain in 34 patients, microneurography may still hold promise in identifying irritable nociceptor phenotypes for future drug trials in painful polyneuropathy.

Selvarajah *et al.* (2019)^74^ demonstrated a relationship between brain volume and functional changes in the somatosensory cortex correlating with the severity of peripheral neuropathy as demonstrated by functional magnetic resonance imaging (fMRI)^75^. Patients with the greatest severity of neuropathy characterized by NCS correlated with the greatest reduction in sensory cortical thickness as well as a widening of the S1 functional representation of the foot and thigh, suggesting deafferentation affecting the sensory neurons with the recruitment of nearby functioning neurons^74^.

In 2020, Wilkinson *et al.*^76^ examined the impact of IV lidocaine on pain severity, sensory phenotype (hyperalgesia vs. sensory loss), and fMRI somatosensory cortical response in 29 patients with DPN compared with 26 healthy controls^74^. Responders were defined by a 30% decrease in pain intensity, lasting for at least 3 weeks^74^. Patients with an irritable nociceptor phenotype were more likely to respond to IV lidocaine than patients with a nonirritable nociceptor phenotype^74^. fMRI in nonresponders had lower S1 cortical volumes and functional connectivity compared with responders and healthy controls^74^. These results suggest that pain phenotypes can help predict treatment response (that is, patients with an irritable nociceptor phenotype are more likely to respond to sodium channel blockers) and that treatment response may be characterized by connectivity between primary somatosensory cortex on fMRI.

## Genetic modifiers of disease risk

A genome-wide association study of the ACCORD and BARI 2D cohorts^77^ found a genetic locus on Chr2q24, which was more frequent in diabetic patients without peripheral neuropathy than in patients with diabetic neuropathy. This is a novel locus associated with the risk of diabetic peripheral neuropathy, and its function has not yet been clearly defined^77^. It has been suggested to be associated with higher tibial nerve expression of the *SCN2A* gene, which is located nearby as well as potentially influencing glucose metabolism and insulin resistance^78^. microRNAs, which regulate up to 30% of human genes, are emerging from animal models of neuropathy risk as possible biomarkers of risk and pathogenesis in diabetic neuropathy in humans^79^. Diabetic sensory neurons demonstrate a unique pattern of microRNA alterations in preclinical models of neuropathy in type 1 diabetes^80^. In type 2 diabetic neuropathy, epigenomic factors such as DNA methylation and post-translational histone modifications are considered possible contributors to the development of “metabolic memory” and risks of developing diabetic complications^81^.

In recent years, a mechanistic model of axonal degeneration demonstrated that the balance between pro-survival factors and pro-degenerative molecules drives axonal metabolism and self-destruction. SARM1 is a pro-degenerative molecule and represents a key step in a program for axonal degeneration following injury (“Wallerian degeneration”)^82^. This pathway was discovered when a colony of C57Bl/6J mice spontaneously developed the *WldS* mutation, which resulted in dramatically slowed Wallerian degeneration^83^. *WldS* mice are resistant to axonal degeneration due to nerve injury and other neuropathic insults, including neurotoxic chemotherapy^84,85^. Subsequently, SARM1 was noted to be required for axonal degeneration in fruit flies and mice^86^. Structure and functional studies showed that SARM1 is a critical enzyme in initiating axonal death^87^. Upstream from SARM1 activity, survival factor NMNAT2 is an endogenous enzyme in healthy axons and restrains SARM1 degenerative activity^88^. The absence of NMNAT2 induced axonal degeneration, but since this pathway is dependent on SARM1 activity^88^, degeneration could be prevented in preclinical models by knocking down SARM1 or inhibiting its function pharmacologically^89^. Deletion of *SARM1* prevented the development of neuropathy in the streptozotocin mouse model of type 1 DPN^90^. SARM1 is emerging as a potential treatment target for multiple forms of neuropathy. It is also possible that genetic variation in *SARM1* can predict disease risk^91^. While data are not available for DPN, recent studies suggest that naturally occurring variation in the *SARM1* gene may increase the risk of amyotrophic lateral sclerosis^91,92^.

## Recent therapeutic developments for DPN

An evolving literature supports the utility of lifestyle-based therapies for patients with DPN and neuropathy associated with prediabetes (Table 2). Short-term exercise trials have demonstrated improvement in gait, strength, and function in small cohorts^58,93–100^. Low-intensity exercises improved quality of life as well as reduced pain and tingling symptoms^101^. Indeed, in diabetic patients without neuropathy, weekly exercise for one year significantly increased distal IENFD compared with those without exercise, suggesting that presymptomatic injury to small unmyelinated fibers may be reversible^102^. Similar results were demonstrated in patients with metabolic syndrome without clinical neuropathy^103^. A clinical trial examining the combination of bariatric surgery and high-intensity exercise in the prevention and treatment of diabetic neuropathy is underway (NCT03617185).

**Table 2.** Summary of exercise, molecular therapeutics, and neuromodulation clinical trials in diabetic polyneuropathy.

	Design	Outcome
**Exercise**		
Proprioceptive exercise training^94^	Prospective study. 8 weeks of proprioceptive exercise (n = 14) vs. control (n = 14)	Improved balance, six-minute walking test (6MWT), Beck Anxiety Inventory, Hamilton Depression Rating scale
Sensorimotor training in middle-aged and older adults^95^	Randomized control trial (RCT). 8 weeks of sensorimotor training (n = 22) vs. usual care only (n = 22)	Velocity, stride length, stance, double limb support improved in test group
Hand, finger, foot exercises^96^	RCT. 8 weeks of exercises (n = 51) vs. control (n = 53)	Motor score and activities of daily living (ADLs) were improved in the test group at 8 and 16 weeks
Combined training (resistance-aerobic)^97^	RCT. 8 weeks of exercise (n = 12) vs. control (n = 12)	No significant change in serum kinesin-1 level, aerobic endurance, upper body strength. Increased lower body strength in test group
Proprioceptive training^98^	Prospective study. 8 weeks of exercise (n = 19) vs. control (n = 19)	Improved balance in exercise group
Strength and balance training^100^	RCT. 8 weeks of intervention (n = 70) vs. control (n = 73)	No changes in health-related quality of life (HrQoL) improved functional status and balance at 6 months in test group
Lifestyle intervention^58^	Prospective study. 12 weeks of a lifestyle intervention (n = 40) vs. control	Diabetic peripheral polyneuropathy (DPN) severity decreased at end of 12 weeks in test group
Exercise vs. lifestyle^102^	Prospective study. 1 year of lifestyle counseling (n = 40) vs. weekly exercise (n = 60)	Distal leg intraepidermal nerve fiber density (IENFD) increased in the exercise cohort vs. no change in the counseling cohort
Exercise + lifestyle counseling^103^	Prospective study. 4 months of intervention (n = 36) vs. control (n = 31)	30-day distal leg reinnervation after capsaicin-induced axotomy is improved in the test group
**Supplements/molecular**		
alpha-lipoic acid^105^	Prospective study. 600 mg daily of alpha-lipoic acid ×40 days (n = 72), no control	50% of patients reported improvement in neuropathic symptoms and improved quality of life
Vitamin E (VENUS)^106^	Double-blind RCT. 12 months of 200 mg mixed tocotrienols twice a day (BID) (n = 150) vs. placebo (n = 150)	No difference in patient-reported total symptom score (TSS), neuropathy impairment score (NIS), or sensory nerve conduction study (NCS)
Vitamin E^107^	Double-blind RCT. 8 weeks of 200 mg tocotrienols BID (n = 39) vs. placebo (41)	Improved NCS conduction velocity (CV), increased nerve growth factor (NGF) levels in test group
Vitamin D_3_^129^	Prospective study. 12 weeks of 50,000 IU vitamin D_3_ weekly (n = 60), no placebo	Improved A1c, vitamin D, Michigan Neuropathy Screening Instrument (MNSI) (questionnaire and exam) compared with baseline
Vitamin D^130^	Prospective study. Single intramuscular (IM) dose of vitamin D 600,000 IU (n = 143), no placebo	Reduced positive symptoms on DN4, total pain score, short-form McGill Pain Questionnaire (SFMPQ) at 20 weeks vs. baseline
Vitamin D^131^	Prospective study. Vitamin D supplementation to correct vitamin D deficiency (n = 51), no placebo	Reduced pain scores at 3 months vs. baseline
Vitamin D^132^	Prospective study. 8 weeks of weekly vitamin D supplementation (n = 57) vs. placebo (n = 55)	Neuropathy Symptom Score (NSS) improved but no difference in Neuropathy Disability Score (NDS) or NCS
EMA401 (EMPADINE)^109^	RCT. 12 weeks of (EMA401) 100 mg BID (n = 137)	Prematurely terminated because of hepatotoxicity
Nav1.7 blocker PF-05089771^110^	RCT. 4 weeks of PF-05089771 150 mg BID (n = 40), pregabalin 150 mg BID (n = 40), placebo (n = 40)	No significant change in pain score of PF-05089771 vs. placebo; pain score of pregabalin was improved vs. placebo
Benfotiamine^126^	RCT. 6 weeks of benfotiamine 600 mg daily (n = 47) vs. 300 mg daily (n = 45) vs. placebo (n = 41)	Improved in NSS, no difference in TSS
**Neuromodulation**		
High-frequency (10-kHz) spinal cord stimulation^111^	RCT. 6 months of 10-kHz spinal cord stimulation (SCS) in refractory painful diabetic neuropathy (n = 95) vs. control (n = 94)	75/95 in intervention arm (vs. 5/94 in control arm) achieved 50% pain reduction without neurological worsening
Spinal cord stimulation, long-term (5-year follow-up)^112^	Prospective study. 5 years of spinal cord stimulator (N = 48), no control.	55% of patients achieved 50% pain reduction. Mean duration of treatment = 60 months 80% of patients still used SCS after 5 years
Frequency-modulated electromagnetic neural stimulation (FREMS)^117^	Double-blind RCT. Two series of ten treatments of FREMS or placebo in random sequence, each no more than 3 weeks. n = 31	Reduced daytime and nighttime pain score, increased sensory perception (monofilament, vibration), and motor NCS at end of treatment and at 4 months follow-up
Group acupuncture^121^	RCT. 12 weeks of twice-weekly group acupuncture (n = 14) vs. once-weekly acupuncture (n = 14) vs. usual care (n = 14)	Decreased weekly pain intensity at week 12 vs. baseline; results were not maintained after acupuncture ended

In the past few years, a handful of small cohort clinical trials^104^ have explored the effects of vitamin supplementation in diabetic neuropathy: oral alpha lipoic acid^105^, vitamin E^106,107^, vitamin D^108^, EMA401^109^, and sodium channel blocker PF-05089771^110^; these studies have mostly been negative or inconclusive.

Several recent studies have explored neuromodulation as a therapeutic strategy for painful DPN. In 2021, results from the SENZA-PDN trial (multicenter, randomized comparison of conventional medical management against 10-kHz spinal cord stimulation plus medical management) showed significant pain relief sustained over 6 months^111^ in 95 patients in the treatment group. Long-term follow-up results from a smaller trial were published in 2018, noting treatment success in 55% of spinal cord stimulation in patients with painful diabetic neuropathy after 5 years^112^. Patients with more severe neuropathy (Michigan Diabetic Neuropathy Score [MDNS] 3) had a higher risk of long-term treatment failure at the 5-year follow-up, resulting in device removal^112^. In contrast, a higher baseline nocturnal pain score was associated with decreased risk of treatment failure^112^. Complications include infection (5%) and pocket pain (25%), and about half required adjustments or replacements in equipment, such as lead revision (10%) or battery replacement (33%)^112^.

Attempts at peripheral neurostimulation have been largely unsuccessful. Bioelectronic therapies were explored in small cohorts of patients with painful diabetic neuropathy: transcutaneous electrical nerve stimulation (TENS) in a retrospective study showed an average use of 1.7 years, with 76% of patients reporting subjective improvement in pain^113,114^. Pulse-dose electrical stimulation for 4 weeks in 10 patients showed a reduction in pain at the end of treatment and for 4 weeks after discontinuation of treatment^115,116^. Frequency-modulated electromagnetic neural stimulation (FREMS) showed pain reduction and increased tactile perception^117^. Pulsed electromagnetic field therapy in painful diabetic neuropathy showed mixed results^118–120^. Acupuncture showed a decrease in pain during a 12-week intervention period, but this response was not sustained after treatment stopped^121^. The ACUDPN trial (NCT03755960) started in 2018 and examined the effect of acupuncture over 8 weeks on pain severity and nerve conduction parameters; results are pending.

The American Academy of Neurology updated guidelines for the management of painful DPN in 2022^122^. Four classes of medications are recommended in the treatment of painful DPN: gabapentinoids, tricyclic antidepressants, sodium channel blockers, and serotonin and norepinephrine reuptake inhibitors; opioids are not recommended^122^. If a trial of one medication class achieves partial pain control, adding on a second class is recommended. If one class has no effect on pain control, a trial of a different medication class is recommended over attempting a second agent from the same class. Clarifying expectations of pain management is essential in achieving patient satisfaction: while patients often expect complete pain resolution, a 30% reduction in pain level is considered successful in clinical trials, and the goal of pharmacotherapy is to reduce but not necessarily eliminate neuropathic pain^122^. Evaluation for comorbidities such as sleep and mood disorders is recommended. These are more prevalent than in the general population, and both affect pain experience, and treatment can be more effective in improving pain control and quality of life^122^.

A number of potentially disease-modifying therapies are in preclinical development. Sirtuins such as resveratrol^123^ have been suggested as potential pharmacologic targets for the prevention of diabetic neuropathy, given their role in off-loading mitochondrial respiratory demand. Benfotiamine was explored as a potential therapy via reducing excess glucose metabolism down the pentose phosphate pathway, which in turn forms advanced glycation end-products, resulting in increased oxidative stress^124^. A 3-week placebo-controlled trial in 2005 showed a reduction in neuropathic pain but no improvement in vibratory sensation^125^. This was confirmed in a phase III trial in 2008, and long-term benfotiamine supplementation showed improvement in NCSs and inflammatory markers such as TNF-α, IL-6, and IL-18^126^. A small study has been proposed to evaluate the effect of benfotiamine on IENFD and diabetic neuropathy (NCT01868191). Omega-3 polyunsaturated fatty acids are also of interest in preventing the progression of neuropathy with early preclinical evidence of preserving nerve function^127^, and multiple clinical trials (NCT05169060, NCT05145452, and NCT04222660) are under way. Early Phase Pain Investigation Clinical Network (EPPIC-Net) is currently funding two clinical trials for DPN: the development of NRD135S. E1, a nonopioid oral analgesic that downregulates purinergic receptors involved in the central nervous system processing of pain (NCT02345291), and a phase 2 study on the efficacy of topical pirenzepine, a muscarinic antagonist which showed promise in mouse models for painful DPN (NCT04786340)^128^.

## Conclusions

DPN is a highly prevalent disorder associated with significant patient morbidity and healthcare costs. While there is an urgent need for more *effective* symptomatic treatments targeting neuropathic pain and disease-modifying and preventative therapies, recent advances promise to accelerate therapeutic development. Refined diagnostic criteria and characterization of specific pain phenotypes will inform clinical trial design, and developing biomarkers promise to facilitate earlier diagnosis and design of clinical trials for patients early in the disease course. Preclinical studies of metabolic and genetic risks for neuropathy are being translated into clinical trials.
